# Protease-Activated Receptor (PAR)2, but Not PAR1, Is Involved in Collateral Formation and Anti-Inflammatory Monocyte Polarization in a Mouse Hind Limb Ischemia Model

**DOI:** 10.1371/journal.pone.0061923

**Published:** 2013-04-18

**Authors:** Lisa G. van den Hengel, Alwine A. Hellingman, Anne Yael Nossent, Annemarie M. van Oeveren-Rietdijk, Margreet R. de Vries, C. Arnold Spek, Anton Jan van Zonneveld, Pieter H. Reitsma, Jaap F. Hamming, Hetty C. de Boer, Henri H. Versteeg, Paul H. A. Quax

**Affiliations:** 1 Einthoven Laboratory for Experimental Vascular Medicine, Leiden University Medical Center, Leiden, The Netherlands; 2 Department of Vascular Surgery, Leiden University Medical Center, Leiden, The Netherlands; 3 Department of Nephrology, Leiden University Medical Center, Leiden, The Netherlands; 4 Center for Experimental and Molecular Medicine, Academic Medical Center, University of Amsterdam, Amsterdam, The Netherlands; Bristol Heart Institute, University of Bristol, United States of America

## Abstract

**Aims:**

In collateral development (i.e. arteriogenesis), mononuclear cells are important and exist as a heterogeneous population consisting of pro-inflammatory and anti-inflammatory/repair-associated cells. Protease-activated receptor (PAR)1 and PAR2 are G-protein-coupled receptors that are both expressed by mononuclear cells and are involved in pro-inflammatory reactions, while PAR2 also plays a role in repair-associated responses. Here, we investigated the physiological role of PAR1 and PAR2 in arteriogenesis in a murine hind limb ischemia model.

**Methods and Results:**

PAR1-deficient (PAR1-/-), PAR2-deficient (PAR2-/-) and wild-type (WT) mice underwent femoral artery ligation. Laser Doppler measurements revealed reduced post-ischemic blood flow recovery in PAR2-/- hind limbs when compared to WT, while PAR1-/- mice were not affected. Upon ischemia, reduced numbers of smooth muscle actin (SMA)-positive collaterals and CD31-positive capillaries were found in PAR2-/- mice when compared to WT mice, whereas these parameters in PAR1-/- mice did not differ from WT mice. The pool of circulating repair-associated (Ly6C-low) monocytes and the number of repair-associated (CD206-positive) macrophages surrounding collaterals in the hind limbs were increased in WT and PAR1-/- mice, but unaffected in PAR2-/- mice. The number of repair-associated macrophages in PAR2-/- hind limbs correlated with CD11b- and CD115-expression on the circulating monocytes in these animals, suggesting that monocyte extravasation and M-CSF-dependent differentiation into repair-associated cells are hampered.

**Conclusion:**

PAR2, but not PAR1, is involved in arteriogenesis and promotes the repair-associated response in ischemic tissues. Therefore, PAR2 potentially forms a new pro-arteriogenic target in coronary artery disease (CAD) patients.

## Introduction

Cardiovascular disease is one of the world's leading causes of mortality. Occlusion of coronary arteries or large peripheral arteries, as a consequence of an atherosclerotic lesion or thrombus, causes insufficient blood supply to the heart or lower extremities. In response to the resulting increased shear flow, the interconnecting arterioles between the large vessels remodel into mature collaterals, a process referred to as arteriogenesis [1]. However, risk factors related to diabetes or hyperlipidaemia impair this compensatory mechanism [2]. Thus, the discovery of new targets remains instrumental in the development of therapeutic strategies to promote arteriogenesis.

At the onset of arteriogenesis, increased shear stress against the inner arteriolar wall facilitates endothelium-dependent attraction, adhesion and extravasation of circulating monocytes toward the pre-existing arterioles. As a consequence, monocytes differentiate into macrophages followed by secretion of a variety of arteriogenic cytokines, including vascular growth factors, matrix-degrading proteases and chemo-attractants, which support collateral maturation [3].

Monocytes and macrophages are pivotal players in this remodelling process [4]. Monocytes are a heterogeneous population of mononuclear cells from which two main functionally different subsets have been identified that are distinguished by the expression of the chemokine receptors CX3CR1 and CCR2, as well as the hematopoietic differentiation antigen Ly6C[5]. The Ly6C-high monocytes infiltrate in inflamed tissue in a CCR2-dependent fashion to mediate the progression of the inflammatory response. The population of Ly6C-low monocytes mainly exhibits a patrolling character that is dependent on CX3CR1-mediated intravascular adhesion, and when differentiated into macrophages, participates in the anti-inflammatory/repair-associated response in order to support wound healing and tissue remodelling [5], [6].

Only few studies provide evidence on the implication of each mononuclear subset in arteriogenesis. Blood flow recovery was improved in the presence of pro-inflammatory monocytes rather than the resident circulating population [7], [8], however, others have indicated the support of repair-associated macrophages in the vascular remodelling process [9]. It therefore remains controversial which mononuclear population is the major contributor in collateral remodelling.

Protease-activated receptors (PARs) are a family of G-protein-coupled receptors comprising four members, of which PAR1 and PAR2 are the best characterized. Protease-mediated cleavage of the PAR N-terminus results in downstream G-protein-coupled signalling. While PAR1 and PAR2 belong to the same receptor family, these PARs initiate distinct cellular processes that may result from coupling to distinct G-proteins and activation by different proteases. PAR1 is activated by the coagulation factors thrombin and factor Xa (fXa), endothelial protein C receptor (EPCR)-bound activated protein C (APC), matrix metalloproteinases (MMPs) and plasmin, whereas trypsin, mast cell tryptase, fXa and the coagulation factor complex tissue factor (TF):factor VIIa (fVIIa) cleave PAR2 [10].

PAR1 and PAR2 are both expressed on peripheral monocytes and remain functionally present after differentiation into macrophages [11]. Both receptors induce leukocyte recruitment including the sequential events of rolling, adhesion and migration, suggesting that these signalling receptors may play important roles in monocyte-driven collateral formation [12], [13]. While the functions of both PAR1 and PAR2 are involved in inflammatory processes as demonstrated in different animal disease models [14]–[16], only PAR2 function has been implicated in the repair-associated response. In a mouse model of experimentally-induced colitis, PAR2 activation reduces the T-helper cell type 1 response by inhibition of pro-inflammatory cytokine production and thereby promotes survival [17]. In addition, PAR2 has also been shown to mediate the differentiation of macrophages into an anti-inflammatory phenotype, pointing towards a possible contribution of PAR2 in the growth of collaterals [18].

Earlier studies have considered PAR1 and PAR2 as potential therapeutic targets in the promotion of arteriogenesis. Administration of thrombin, trypsin or a PAR2-derived synthetic peptide enhances blood flow recovery, but only for thrombin it is known that it increases collateral formation after induction of hind limb ischemia by excision of the femoral artery [19], [20]. While in these studies collateral formation was dependent on supraphysiological concentrations of the PAR1-agonist thrombin and detailed data on collateralization upon PAR2 activation is missing, direct evidence for a physiological role of PAR1 and PAR2 in collateral formation is still lacking. A role for PAR2 in angiogenesis, however, has clearly been demonstrated using the PAR2 derived synthetic peptide [20], but also by the reported direct effects of PAR2 on VEGF production [21]–[23].

In the present study, we address the physiological relevance of PAR1 and PAR2 in arteriogenesis in a hind limb ischemia model using mice deficient in either of these receptors. We observed that PAR2, but not PAR1, was involved in the arteriogenic process. Furthermore, while the repair-associated response in the circulation and in the hind limbs was promoted upon ischemia in wild-type (WT) and PAR1-deficient (PAR1-/-) mice, these events were impaired in PAR2-deficient (PAR2-/-) mice.

## Methods

### Hind limb ischemia model

The animal welfare committee of Leiden University Medical Centre approved the *in vivo* experiments (permit number 08–152). Hind limb ischemia was induced in age-matched WT, PAR1-/- and PAR2-/- male mice on a C57Bl6 background. Male PAR1-/- and PAR2-/- mice [24], [25] were originally provided by Jackson Laboratories (Maine, USA) and bred at the animal care facility of the Academic Medical Centre. WT control mice were also purchased from Jackson Laboratories. Mice were typically between 11 and 13 weeks of age at the time of the procedure and had an average weight of 25 gr. Mice were anesthetized with an intraperitoneal injection containing 5 mg/kg Midazolam (Roche, Basel, Switzerland), 0.5 mg/kg Medetomidine (Orion, Helsinki, Finland) and 0.05 mg/kg Fentanyl (Janssen, Geel, Belgium). The femoral artery was dissected free from the nerve and vein and ischemia was induced proximal to the superficial epigastric artery by unilateral single electro-coagulation [26]. The skin was subsequently closed with 6-0 Ethilon sutures. Laser Doppler measurements, angiography and FACS analysis were performed in three consecutive *in vivo* experiments. Furthermore, the mice were analyzed for necrotic toes, but no signs of toe necrosis were observed throughout the study.

### Laser Doppler Perfusion Imaging

Laser Doppler Perfusion Imaging (Moor Instruments, Devon, UK) was used to measure blood flow in the (non-)ischemic paws of PAR1-/- or PAR2-/- and control mice prior to ligation, directly after and subsequently 3, 7, 14, 21 and 28 days after ligation of the femoral artery [26]. Blood flow recovery per mouse was expressed as the perfusion ratio of the ischemic/non-ischemic limb.

### Immunohistochemical staining of paraffin sections

Four weeks after the induction of hind limb ischemia, animals were sacrificed and calf and adductor muscles from (non-)ischemic paws were excised. Muscle tissue was fixated in 4% formaldehyde, embedded in paraffin and cut into five µm sections. Paraffin sections were rehydrated and blocked in methanol containing 0.3% hydrogen peroxide. Collaterals in the adductor muscles were detected with an antibody against smooth muscle actin (SMA, clone 1A4, 1∶750, DakoCytomation, Glostrup, Denmark). For staining of capillaries in calf muscles, antigen retrieval was performed with trypsin followed by the detection of endothelial cells using a CD31 antibody (clone 390, 1∶200, BD Biosciences, San Jose, CA, USA) of which the stain intensity was amplified with avidin-biotin-peroxidase complex (DakoCytomation). Finally, all specimens were stained with Nova Red (Vector laboratories, Burlingame, CA, USA) and counterstained with haematoxylin solution. Quantification was performed by counting the number of SMA-positive and CD31-positive objects in nine fields (10× magnification) per mouse using ImageJ software.

### Mouse aortic sprouting assay

Mouse thoracic aortas were isolated from WT and PAR2-/- mice. Peri-aortic adipose tissues were carefully removed and the aortas were subsequently cut into thin rings, embedded in matrigel, and covered with endothelial basal medium (EBM; Lonza, Basel, Switzerland) containing 2% serum and penicillin/streptomycin. Aortic rings were kept at 37°C and the number of endothelial sprouts were counted on day 5.

### Pre-existing pial collateral density

Measurement of pial collateral density was performed as previously described [27]. In short, animals were systemically heparinized and anesthetized using ketamine (100 mg/kg) and xylazine (10 mg/kg) prior to vascular casting. Maximal dilation was accomplished by canulation of the thoracic aorta and infusion of nitroprusside (30 µg/ml) and papaverine (40 µg/ml) in PBS at 100 mmHg for 3 minutes. Fixation of the dorsal cerebral circulation was accomplished by removal of the calvarium and dura mater, followed by topical application of 4% paraformaldehyde. Yellow Microfil™ (Flow Tech Inc.) with viscosity sufficient to prevent capillary filling was infused under a stereomicroscope. Brains were incubated in Evans Blue (2 µg/ml) for several days prior to imaging in order to provide contrast for improved visualization of yellow Microfil™. Collateral density was calculated in PAR2^−/−^ and WT mice by determining the total number of pial collaterals and dividing by the dorsal surface area of the cerebral hemispheres. Areas were excluded when they were damaged, had poor filling with Microfil™, or were otherwise uncountable.

### Flow cytometry

Before and seven days after femoral artery ligation, peripheral blood was drawn via the tail vein from WT, PAR1-/- and PAR2-/- mice, anti-coagulated with EDTA. For FACS analysis, blood was incubated with a heterogeneous mix of fluorescent antibodies against B220-APC-Cy7 (eBioscience, San Diego, CA, USA), CD11b conjugated with APC (Biolegend, San Diego, CA, USA), Ly6G-PE (Becton Dickinson, Franklin Lakes, New Jersey, USA), CD115-PerCP-Cy5.5 (R&D Systems, Minneapolis, MN, USA) and Ly6C-FITC (Bioconnect, Huissen, The Netherlands). After labelling, cell suspensions were washed with PBS containing 1% bovine serum albumin (BSA, Sigma-Aldrich, St. Louis, USA) and 0.01% sodium azide. Erythrocytes were removed by addition of lysis buffer (0.155 M NH_4_Cl, 0.01 M KHCO_3_, 0.1 mM EDTA) and finally, cells were fixed with 1% paraformaldehyde. Monocytes were gated based on their expression profile: CD11b-positive, Ly6G-negative and CD115-positive (Supplementary material online, Figure S1). Pro-inflammatory and repair-associated monocytes were identified based on high or low expression levels of Ly6C, respectively.

### Immunohistochemical staining of frozen sections

One week after ligation, (non-)ischemic hind limb adductor muscles were excised from WT and PAR2-/- mice and directly frozen in liquid nitrogen. The adductor muscles were cut into 6 µm sections which were then fixated in ice-cold acetone. Specimens were blocked with PBS containing 2% fetal calf serum (FCS, PAA Laboratories, Cölbe, Germany) and 3% BSA followed by dual staining of arterial vessels and repair-associated macrophages using a Cy3-conjugated SMA antibody (clone 1A4, 1∶1000, Sigma-Aldrich) and a FITC-labelled mannose receptor CD206 antibody (clone MR5D3, 1∶50, AbD Serotec, Oxford, UK), respectively. Sections were covered with DAPI-containing Vectashield mounting medium (Vector Laboratories). Quantification was performed by counting the number of CD206-positive cells surrounding a vessel within an area of 200×200 µm (20× magnification) using Zen 2009 software (Carl Zeiss, Jena, Germany).

### Statistical analysis

Results are expressed as means ± SEM. The repeated measures ANOVA was used to compare means between groups over a repeated series of time. An independent t-test was performed to compare means between selected groups and a paired t-test was used to analyse means of a group between two different time points. Two-sided p-values <0.05 were considered statistically significant. All calculations were performed in SPSS 16.0.

## Results

### PAR2, but not PAR1, contributes to post-ischemic blood flow recovery and collateral formation

**Figure 1:**
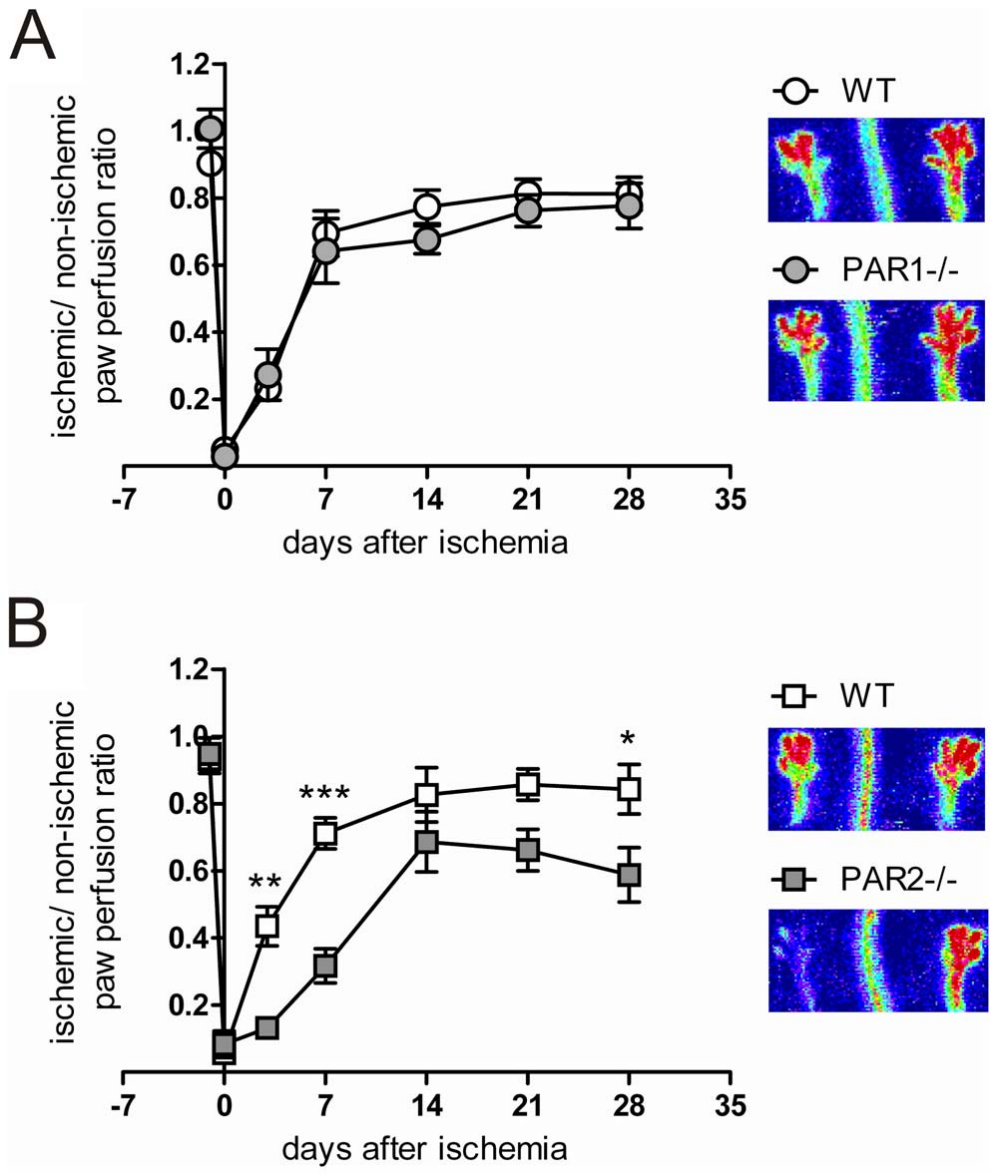
PAR2 mediates blood flow restoration in a hind limb ischemia model. The average blood perfusion ratio between ischemic and non-ischemic hind limbs was measured with laser Doppler before, directly after ligation, at day 3 and every week in a period of four weeks. Representative images of laser Doppler measurements of the ligated hind limbs are shown. (A) The ratio measured in PAR1-/- mice (n = 10) compared to the ratio measured in WT mice (n = 9). (B) The blood perfusion in PAR2-/- mice (n = 7) was compared to the ratio measured in WT mice (n = 10). * p<0.05, ** p<0.01, *** p<0.001.

To explore whether PAR1 and PAR2 are involved in arteriogenesis, we performed a femoral artery ligation and subsequently monitored blood flow by laser Doppler measurements in the hind limbs of WT, PAR1-/- and PAR2-/- mice. Blood flow in the ischemic hind limbs of WT and PAR1-/- mice rapidly restored to ∼70% within 14 days after the femoral artery ligation (Figure 1A). In contrast, PAR2-/- mice showed significantly diminished blood flow recovery after the induction of hind limb ischemia when compared to control mice. Recovery was more than two times reduced after seven days (Figure 1B, p<0.05). The different blood perfusion ratios of WT mice at day 3 as shown in figure 1A and 1B is due to inter-experimental variation. Thus, PAR2 is required to restore blood supply in peripheral tissue after an ischemic event.

**Figure 2:**
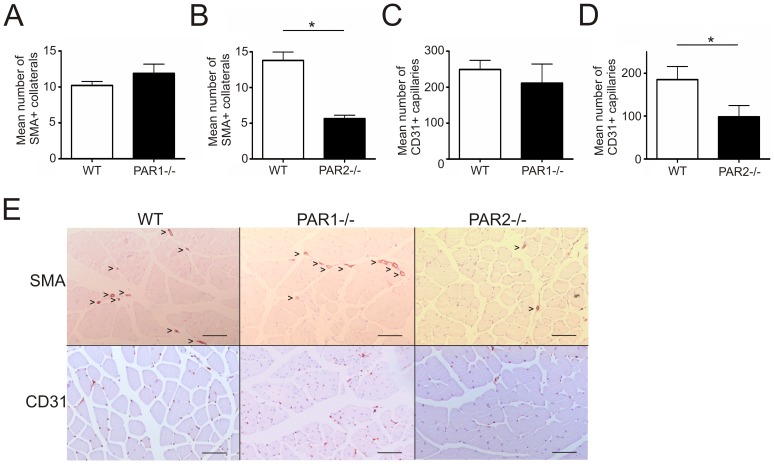
Revascularization upon ischemia is PAR2-dependent. (A) SMA staining was performed to detect collaterals in the ischemic adductor muscles and subsequently quantified. Mean collateral number in WT mice was compared to PAR1-/- mice (B) and PAR2-/- mice. (C) Capillary density was quantified after CD31 staining of ischemic calf muscles. Mean capillary number of WT was compared to PAR1-/- mice (D) and PAR2-/- mice. (E) Images represent the CD31 and SMA stainings of ischemic hind limbs of WT, PAR1-/- and PAR2-/- mice. Arrowheads indicate vessels. Bar, 100 µm. * p<0.05.

Immunohistochemical staining revealed a similar number of SMA-positive collaterals in the post-ischemic adductor muscles of WT and PAR1-/- mice, which is in line with the laser Doppler measurements (Figure 2A). In contrast, PAR2-/- mice showed a significantly reduced number of collaterals in the post-ischemic adductor muscle when compared to control mice (Figure 2B, p<0.05). In addition, the ischemia-induced formation of CD31-positive capillaries in calf muscles of PAR2-/- mice was also decreased when compared to control mice (Figure 2D; p<0.05), whereas the capillary density was not affected in PAR1-/- mice (Figure 2C). The collateral and capillary density in the non-ischemic paw was not different between PAR2-/- mice and WT mice (Supplementary material online, Figure S2). Figure 2E shows typical examples of SMA- and CD31-staining for WT, PAR1-/- and PAR2-/- mice. In addition to the capillary staining, we found reduced sprout formation in PAR2-/- aortas when compared to WT aortas in an *ex vivo* aortic sprouting assay (Supplementary material online, Figure S3), indicating that impaired angiogenesis is a general phenomenon in PAR2-/- mice that is independent of arteriogenesis.

**Figure 3:**
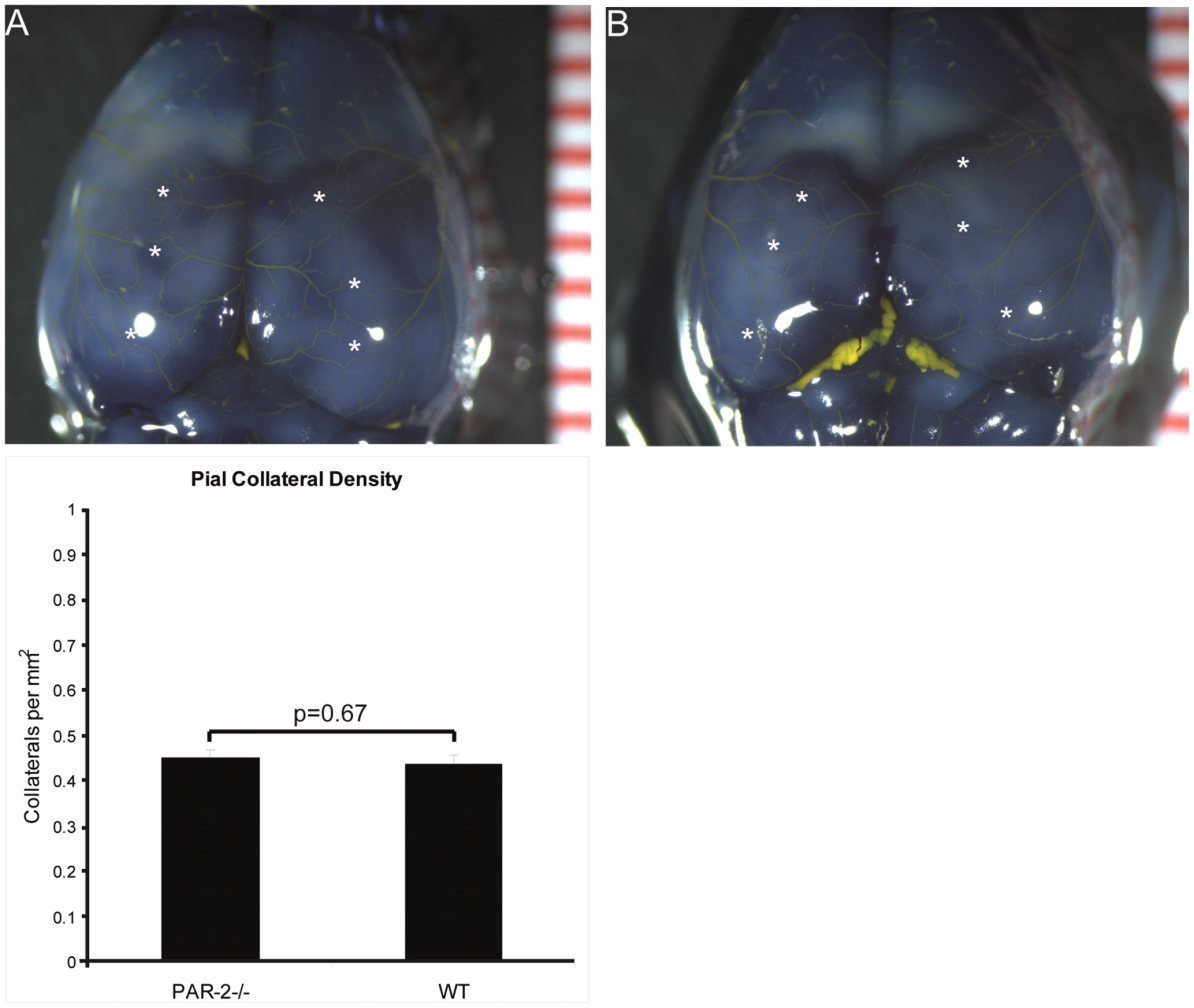
Representative images of the pial circulation in PAR2^-/-^ (A) and WT mice (B). To illustrate, several, but not all collateral arterioles have been indicated with white asterisks. Following exsanguination and maximal dilation of the dorsal cerebral circulation, Microfil™ was used as a casting agent, after which the whole brain was fixated in 4% PFA and subsequently stained in Evans Blue for contrast. (C) Pial collateral density was calculated in PAR2^-/-^ and WT mice by dividing the sum of collaterals between anterior, middle and posterior cerebral arteries by the surface area of the cerebral hemispheres. Areas were excluded when they were damaged, had poor filling with Microfil™, or were otherwise uncountable. All values are presented as the mean ± SEM.

To rule out whether differences in the pre-existing vascular bed contribute to the differences in vascular remodelling as observed, the pial vascular bed of PAR2-/- and wild type mice was visualized and the pre-existing collateral connections were quantified (Figure 3) as described previously [27]. We could not observe any differences between wild type and PAR2-/- mice in the number of pre-existing collateral connections in the pial vascular bed. In conclusion, PAR2 deficiency, but not PAR1 deficiency, resulted in reduced vascular remodelling after an ischemic event, both at the level of angiogenesis and arteriogenesis.

### Ischemia-induced skewing of monocytes towards a repair-associated phenotype is PAR2-dependent

**Figure 4:**
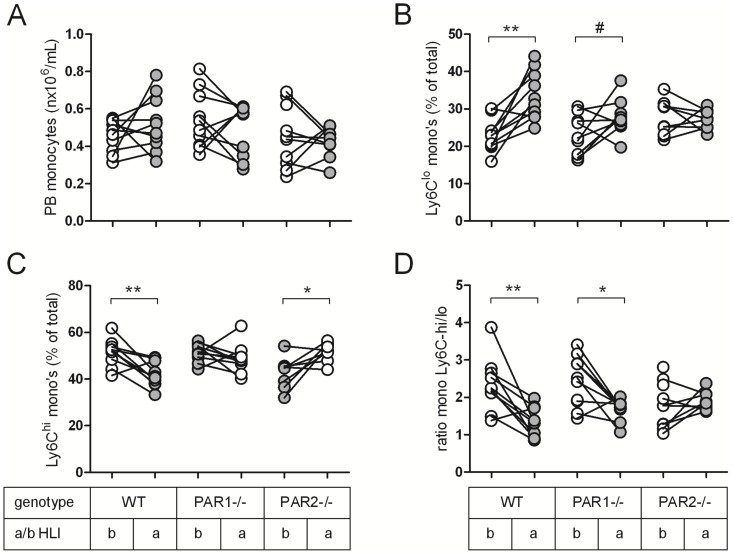
PAR2 contributes to the differentiation of circulating monocytes after femoral artery ligation. Before (b, white circles) and seven days after ligation (a, grey circles), blood was drawn from WT, PAR1-/- and PAR2-/- mice to perform FACS analysis. (A) Absolute number of peripheral blood (PB) monocytes. Monocytes were gated to analyse the change in levels of (B) repair-associated Ly6C-low monocytes and (C) pro-inflammatory Ly6C-high monocytes upon ischemia, which is indicated in percentage of total monocytes. (D) Change in ratio of Ly6C-high/low monocytes upon ligation. # p<0.1, * p<0.05, ** p<0.01.

Since the repair-associated Ly6C-low and pro-inflammatory Ly6C-high subsets of monocytes have been identified with functionally different impacts on processes like atherosclerosis [28], it was of interest to assess which of these subpopulations play a role in PAR2-dependent collateral development. Therefore, we determined alterations in the monocyte subset levels in peripheral blood seven days after ligation, since at that time point the blood flow recovery in the post-ischemic hind limbs of PAR2-/- mice was severely impaired when compared to WT mice (Figure 1B). The absolute number of monocytes per mL whole blood was equal between the three experimental groups before and one week after ligation (Figure 4A). FACS analysis showed an increase in the percentage of Ly6C-low monocytes of total monocytes upon ligation in WT mice (p<0.01) and a trend in PAR1-/- mice (p<0.1), while the percentage of Ly6C-low monocytes in PAR2-/- mice was not changed upon ischemia (Figure 4B). Furthermore, the pool of pro-inflammatory Ly6C-high monocytes was reduced in WT (p<0.01) and, conversely, an increase in this monocyte subset was observed in PAR2-/- mice after ligation (Figure 4C, p<0.05), while PAR1-/- mice showed no difference. To show the change in balance in monocyte subsets in more detail, the relative ratio between Ly6C-high and Ly6C-low monocyte subsets was determined: the ratio was decreased in WT and PAR1-/- mice after ligation (p<0.01 and p<0.05, resp), while this ratio was slightly, but not significantly increased in PAR2-/- mice (Figure 4D). This indicates that PAR-2 is required in the phenotypic switch from pro-inflammatory towards repair-associated monocytes upon ischemia.

### Accumulation of perivascular repair-associated macrophages after ischemia is reduced in PAR2-/- mice

**Figure 5:**
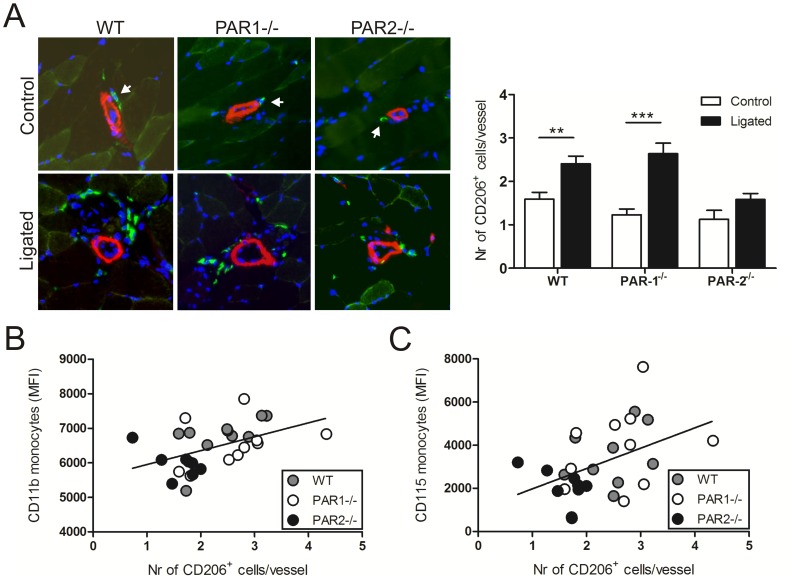
Increased accumulation of repair-associated macrophages surrounding collaterals in ischemic hind limbs is PAR2-dependent. (A) Stainings of CD206-positive macrophages (green) and SMA-positive vessels (red) in non-ischemic (control) and ischemic (ligated) hind limbs of WT, PAR1-/- and PAR2-/- mice are shown. Nuclei were visualized with DAPI (blue). Arrows indicate single macrophages in the non-ischemic adductor. Quantification of the average number of repair-associated macrophages per vessel is indicated on the right. (B) Correlation between the number of CD206-positive macrophages in the ischemic tissues and the expression of CD11b and (C) CD115 on monocytes. ** p<0.01, *** p<0.001.

The impaired ability of PAR2-/- monocytes to be skewed towards an anti-inflammatory phenotype upon ischemia led us to hypothesize that the number of repair-associated macrophages around the developing collaterals may also be diminished in the PAR2-/- post-ischemic muscles seven days after ligation. Femoral artery ligation induced elevated accumulation of repair-associated (CD206-positive) macrophages in the direct vicinity of vessels in the adductor muscles of WT (p<0.01) and PAR1-/- mice (p<0.001); however, the number of invaded CD206-positive macrophages surrounding the PAR2-deficient vascular structures did not change significantly (Figure 5A), which confirms our hypothesis. PAR1 or PAR2 deficiency did not affect the number of CD206-positive macrophages in the non-ischemic hind limbs when compared to WT mice (Figure 5A). Interestingly, PAR2-/- monocytes showed reduced expression of the adhesion receptor CD11b and of CD115 (M-CSF receptor) when compared to WT and PAR1-/- monocytes (Supplementary material online, Figure S4). Since CD11b is important in firm adhesion of myeloid leukocytes to the endothelium [29], lower expression of this β2-integrin may prevent leukocytes to extravasate. Furthermore, diminished expression of CD115 may hamper the differentiation of monocytes into repair-associated macrophages. Indeed, the number of CD206-positive macrophages in the ischemic adductors of WT, PAR1-/- and PAR2-/- mice showed a significant linear regression of CD11b expression (MFI; p = 0.012, r^2^ = 0.221) and CD115 expression (MFI; p = 0.019, r^2^ = 0.195) by the monocytes (Figure 5B,C). Taken together, this indicates that PAR2 was involved in the anti-inflammatory response upon arterial occlusion, which may support the development of new collaterals.

## Discussion

The current study shows that PAR2, and not PAR1, contributes to post-ischemic blood flow restoration and collateral remodelling in a hind limb ischemia model. In parallel, we found a reduced pool of anti-inflammatory mononuclear cells in the circulation and a decreased accumulation of repair-associated macrophages in the post-ischemic hind limbs of PAR2-/- mice upon ischemia.

To investigate the role of PARs in arteriogenesis *in vivo*, we ligated the femoral artery in WT, PAR1-/- and PAR2-/- mice. PAR1-/- mice showed the same peripheral vascularisation and hemodynamic recovery upon induction of hind limb ischemia as WT mice. Although an earlier study showed that the PAR1 agonist thrombin promoted collateral formation in a rabbit hind limb ischemia model [19], we believe that the supraphysiological concentrations of thrombin used in that study suggests a role for PAR4, rather than PAR1, as PAR4 can solely be activated by high levels of thrombin [30]. Alternatively, a PAR-independent mechanism such as thrombin-induced fibrin formation may explain the effect of thrombin on enhanced collateralisation [31].

Our study identified PAR2 as a functional receptor in arteriogenesis, as the arterioles in the post-ischemic hind limbs of PAR2-/- mice failed to remodel into SMA-expressing high-conductance vessels accompanied with impaired blood flow recovery. Similarly, Milia *et al.* [20] showed in mice that the blood flow restoration was increased after induction of hind limb ischemia when PAR2 was exogenously activated by intramuscular administration of synthetic PAR2 peptides or trypsin. PAR2 activation enhanced capillary formation in the ischemic hind limbs, which is in line with the impaired ischemia-induced angiogenic capacity in PAR2-/- mice as observed in our study both *in vivo* in the calf muscle (Figure 2) and *in vitro* in the aortic ring sprouting assay (Figure S3). However, while the report by Milia *et al.* [20] did not provide further information on arteriogenesis and collateral formation in the adductor muscles as a potential vascular remodelling process underlying PAR2-dependent blood flow recovery, our data show that PAR2-deficiency is linked to impaired collateral formation in the adductors in tissue recovery. Interestingly in this respect is a study of Stone et al. demonstrating that overexpression of tissue kallikrein, a known activator of PAR2 signalling, strongly enhances collateral formation [32].

Mononuclear cells form a heterogeneous population of cells and are established players in the onset and maturation stage of arteriogenesis; shear stress-induced endothelial activation causes the attraction and infiltration of monocytes and lymphocytes towards the developing collaterals. Monocytes can be subdivided into two functionally distinct populations, the pro-inflammatory ‘Ly6C-high’ and repair-associated ‘Ly6C-low’ cells, which are both capable of entering the extravascular tissue where they transform into macrophages. We demonstrated that monocytes were skewed towards a repair-associated phenotype in both WT and PAR1-/- mice seven days after ligation, while PAR2-/- monocytes failed to show a phenotypic switch towards repair-associated monocytes. Therefore, PAR2 plays a key role in the polarization of monocytes in arteriogenic settings. Since PAR2 is thought to play a role in T-helper cell type1 activation [17] and we previously demonstrated a role for CD4+Th1 cells in the induction of collateral formation [33], PAR2 may also act on these pathways involved in regulating collateral formation.

However, macrophages in ischemic tissue are critical effectors of arteriole growth. Others have shown a role for the pro-inflammatory subpopulation in arteriogenesis [7], [8]. However, the repair-associated macrophages, which are known to be pro-angiogenic and reparative, are thought to act directly on the formation of collaterals [9]. For that reason and because we observed an increased anti-inflammatory population in the circulation of WT mice after ligation, we specifically focused on the CD206-expressing repair-associated macrophages in tissue. Consistent with the change in levels of anti-inflammatory monocytes upon ischemia, we found more repair-associated macrophages around growing arterioles in the post-ischemic hind limbs of WT and PAR1-/- mice when compared to the non-ligated hind limbs. However, the number of perivascular repair-associated macrophages did not change upon ischemic induction in the hind limbs of PAR2-/- mice. Apparently, alterations in macrophage numbers correlated with the degree of revascularisation and blood perfusion restoration. The fact that repair-associated macrophages produce pro-arteriogenic factors that stimulate SMC proliferation and migration, further supports the importance of these macrophages in arteriogenesis [9].

The change in anti-inflammatory monocyte levels in peripheral blood of WT and PAR2-/- mice after ischemia coincided with the presence of repair-associated macrophages in the extravascular tissue of these mice, suggesting that circulating monocytes were a direct source for the macrophages. Interestingly, we found a correlation between the number of repair-associated macrophages after ligation and the expression levels of the adhesion receptor CD11b and the M-CSF receptor (CD115) on monocytes. This indicates that the adhesive capacity required for extravasation and the capability to transform into repair-associated macrophages in response to M-CSF are important features of monocytes to contribute to the repair response during arteriogenesis. Moreover, these data also implicate a role for PAR2 in the migration and differentiation of monocytes upon ischemia.

Apart from monocytes, the repair-associated macrophages may also derive from tissue-resident macrophages, as was observed before in an ischemic reperfusion model [34]. Because it is known that PAR2 promotes the differentiation of LPS-stimulated macrophages towards an anti-inflammatory phenotype [18], macrophage plasticity is therefore also considered to be a potential contributor to the PAR2-dependent accumulation of repair-associated macrophages. In addition, nuclear factor kappa B (NF-κB) p50 subunit is a pivotal modulator of repair-associated macrophage polarization and, as PAR2 activation enhances NF-κB interaction with DNA, it would be of interest to investigate whether PAR2 signalling affects macrophage plasticity through NF-κB transcriptional activity [35], [36].

Besides being present and active on mononuclear cells, PAR2 also influences proliferation of vascular endothelial cells and smooth muscle cells *in vitro* [37], [38]. Therefore it is plausible that PAR2 additionally facilitates vascular remodelling through stimulation of endothelial or smooth muscle cells.

Additional research is needed to investigate which proteases are responsible for the activation of PAR2 during arteriogenesis. Mast cell tryptase is a potential candidate as the presence of mast cells along the collaterals is required for blood flow recovery after the induction of ischemia [39]. Because mast cells release a variety of (other) pro-arteriogenic factors, exploring the role of mast cell tryptase in arteriogenesis is rather challenging [40]. Tissue kallikrein would also be a very interesting candidate, since previous studies have demonstrated that overexpression of this PAR2 activator strongly enhances collateral formation [32]. Other interesting PAR2 activators are blood coagulation-related factors (fVIIa and fX) which can form complexes with the transmembrane protein tissue factor (TF) at the surface of PAR2-bearing vascular and mononuclear cells [10].

In conclusion, the results of this study indicate that occlusion-induced ischemia is relieved by PAR2-mediated growth of collateral arterioles, which is dependent on the presence of repair-associated macrophages. Therefore, the present study reiterates the importance of PAR2 as a target in the development of therapeutic approaches to this beneficial remodelling process.

## Supporting Information

Figure S1 **Gating strategy for peripheral blood monocyte populations.** A gate is drawn on all cells in a FSC/SSC plot (A) to exclude debris. Of the cells gated in plot A, the expression of CD11b (X-axis) and B220 (Y-axis) is shown in plot B, on which gates are placed on the CD11b^neg^ cells and the CD11b^pos^ cells. The CD11b^pos^ cells are selected in plot C, showing expression of Ly6G (X-axis) and SSC (Y-axis), in which Ly6G^pos^/SSC^hi^ cells represent neutrophilic granulocytes, Ly6G^neg^/SSC^hi^ cells represent eosiniphilic granulocytes and the Ly6G^neg^/SSC^lo^ cells represent the non-granulocytic cells. These latter cells are selected in plot E, showing their expression of CD11b (X-axis) and CD115 (Y-axis): CD11b^hi^/CD115^hi^ cells represent the monocytes and the CD11b^dim^/CD115^neg^ cells represent NK cells. The monocytes gated in plot E are selected in plot F, showing their FSC (X-axis) and expression of Ly6C: Ly6C^hi^ cells represent the pro-inflammatory/classical monocytes, Ly6C^med^ cells represent the intermediate monocytes and Ly6C^lo^ cells represent the anti-inflammatory, pro-angiogenic/repair-associated/non-classical monocytes. The CD11b^neg^ cells gated in plot B are selected in plot D and show their expression of the B-cell marker B220 (X-axis) and Ly6C (Y-axis): B220^neg^/Ly6C^neg^ cells represent the T-cells, B220^pos^/Ly6c^neg^ cells are B-cells, B220^neg^/Ly6C^pos^ cells are activated T-cells and B220^pos^/Ly6C^pos^ cells are plasmacytoid dendritic cells (pDCs). (G) shows a summary of all characterized subpopulations.
(TIF)

Figure S2 **Quantification of the number of collaterals and capillaries in the non-ischemic hind limbs of WT and PAR2-/- mice.** (A) Mean number of SMA-positive collaterals in the non-ischemic adductor muscles of WT mice was compared to PAR2-/- mice. (B) Mean CD31-positive capillary density in the non-ischemic calf muscles of WT mice was compared to PAR2-/- mice.
(TIF)

Figure S3 **Quantification of the number of endothelial sprouts in WT and PAR2-/- aortas.** Aortic ring assay was performed with aortas from WT and PAR2-/- mice. Number of endothelial sprouts were counted and mean number (#) of sprouts in WT aortas were compared to the number of sprouts in PAR2-/- aortas.
(TIF)

Figure S4 **Expression of CD11b on monocytes and granulocytes and CD115 expression on monocytes in WT, PAR1-/- and PAR2-/- mice.** FACS analysis was performed with peripheral blood from WT, PAR1-/- and PAR2-/- mice before ligation. The expression levels (MFI) are shown of (A) CD11b on monocytes (B) CD11b on granulocytes and (C) CD115 on monocytes.
(TIF)
