# Supplementary figures and images for: Protease-Activated Receptor (PAR)2, but Not PAR1, Is Involved in Collateral Formation and Anti-Inflammatory Monocyte Polarization in a Mouse Hind Limb Ischemia Model
Source: PLoS One. 2013 Apr 18;8(4):e61923. doi: 10.1371/journal.pone.0061923 (PMC3630144; doi:10.1371/journal.pone.0061923)

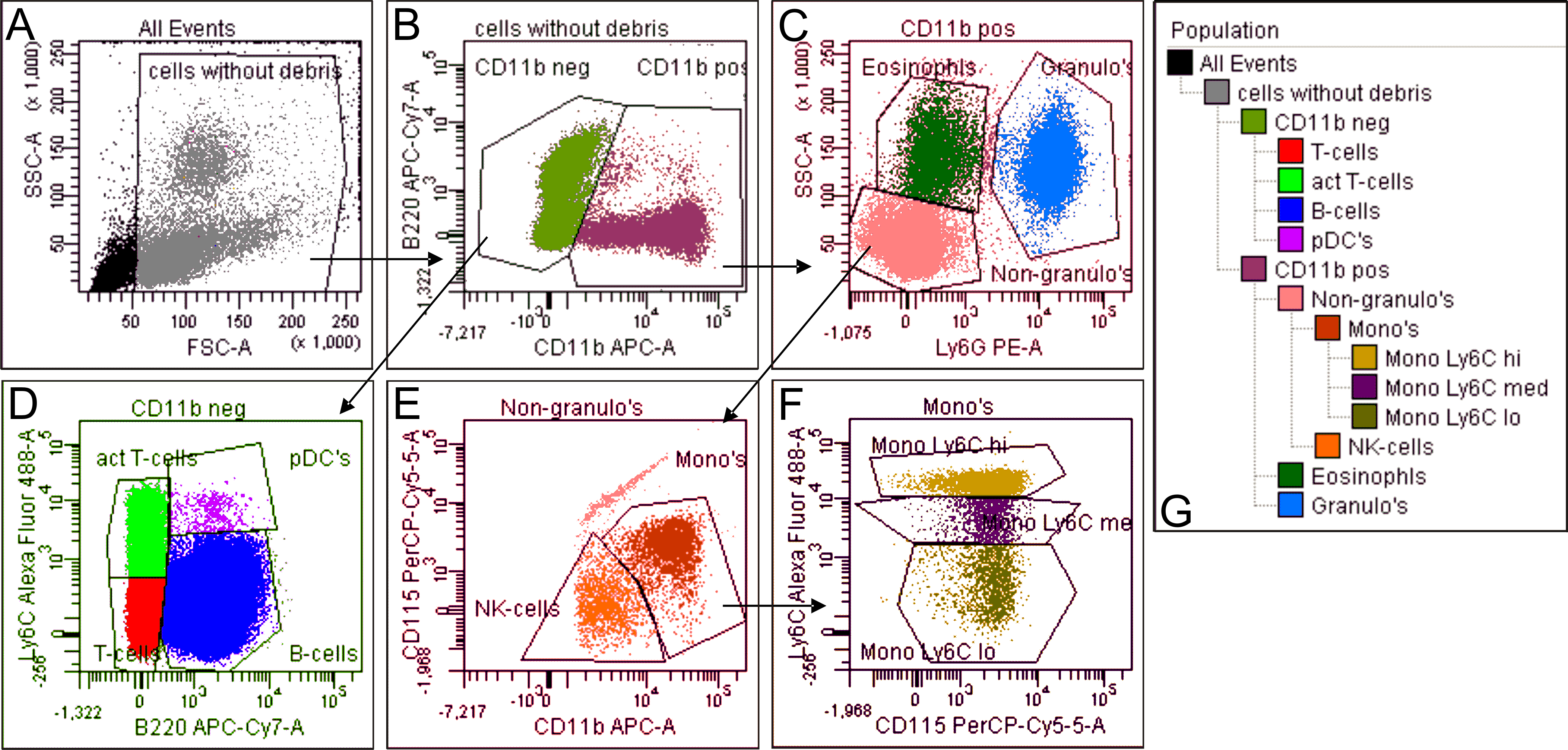

Supplement: Figure S1 — Gating strategy for peripheral blood monocyte populations. A gate is drawn on all cells in a FSC/SSC plot (A) to exclude debris. Of the cells gated in plot A, the expression of CD11b (X-axis) and B220 (Y-axis) is shown in plot B, on which gates are placed on the CD11bneg cells and the CD11bpos cells. The CD11bpos cells are selected in plot C, showing expression of Ly6G (X-axis) and SSC (Y-axis), in which Ly6Gpos/SSChi cells represent neutrophilic granulocytes, Ly6Gneg/SSChi cells represent eosiniphilic granulocytes and the Ly6Gneg/SSClo cells represent the non-granulocytic cells. These latter cells are selected in plot E, showing their expression of CD11b (X-axis) and CD115 (Y-axis): CD11bhi/CD115hi cells represent the monocytes and the CD11bdim/CD115neg cells represent NK cells. The monocytes gated in plot E are selected in plot F, showing their FSC (X-axis) and expression of Ly6C: Ly6Chi cells represent the pro-inflammatory/classical monocytes, Ly6Cmed cells represent the intermediate monocytes and Ly6Clo cells represent the anti-inflammatory, pro-angiogenic/repair-associated/non-classical monocytes. The CD11bneg cells gated in plot B are selected in plot D and show their expression of the B-cell marker B220 (X-axis) and Ly6C (Y-axis): B220neg/Ly6Cneg cells represent the T-cells, B220pos/Ly6cneg cells are B-cells, B220neg/Ly6Cpos cells are activated T-cells and B220pos/Ly6Cpos cells are plasmacytoid dendritic cells (pDCs). (G) shows a summary of all characterized subpopulations. (TIF) [file pone.0061923.s001.tif]

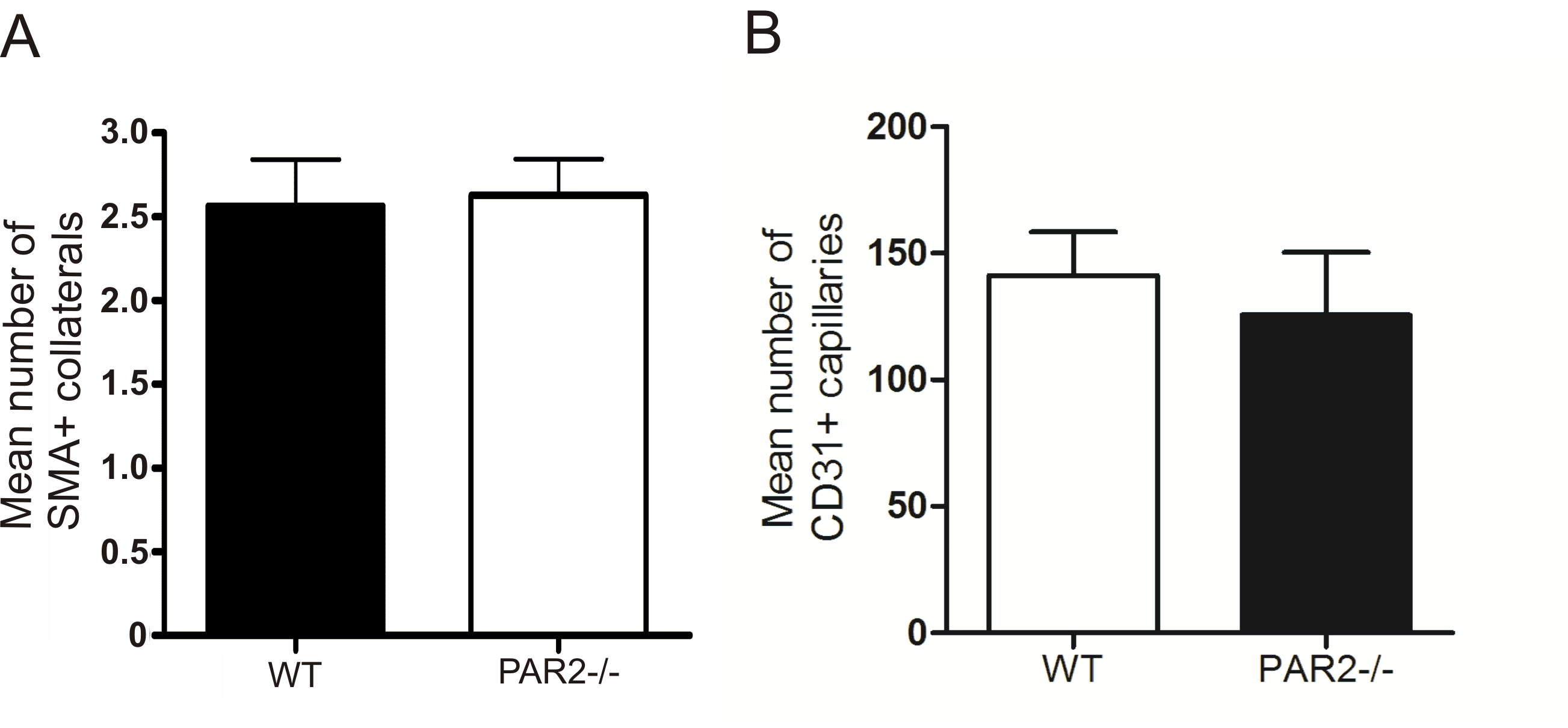

Supplement: Figure S2 — Quantification of the number of collaterals and capillaries in the non-ischemic hind limbs of WT and PAR2-/- mice. (A) Mean number of SMA-positive collaterals in the non-ischemic adductor muscles of WT mice was compared to PAR2-/- mice. (B) Mean CD31-positive capillary density in the non-ischemic calf muscles of WT mice was compared to PAR2-/- mice. (TIF) [file pone.0061923.s002.tif]

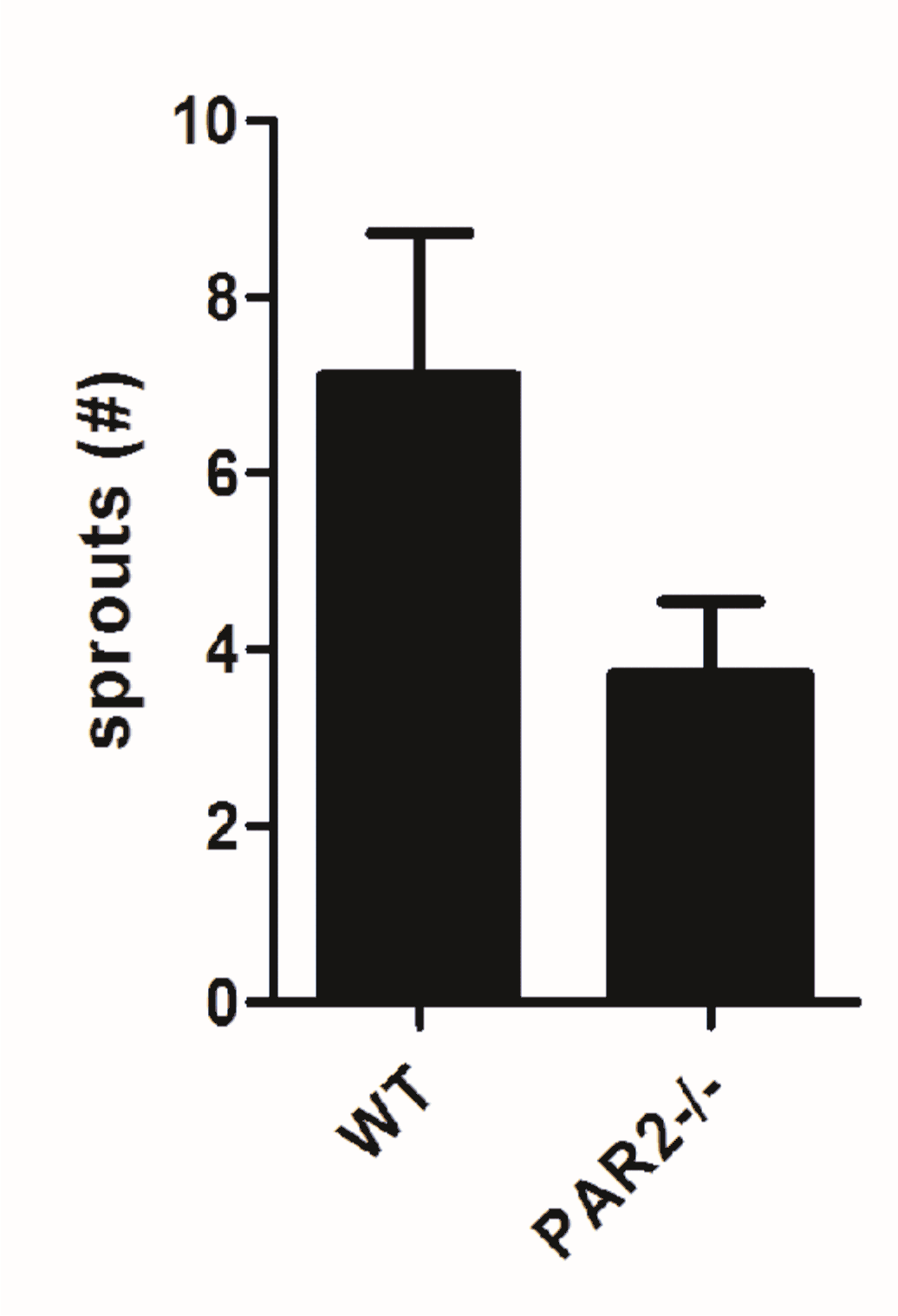

Supplement: Figure S3 — Quantification of the number of endothelial sprouts in WT and PAR2-/- aortas. Aortic ring assay was performed with aortas from WT and PAR2-/- mice. Number of endothelial sprouts were counted and mean number (#) of sprouts in WT aortas were compared to the number of sprouts in PAR2-/- aortas. (TIF) [file pone.0061923.s003.tif]

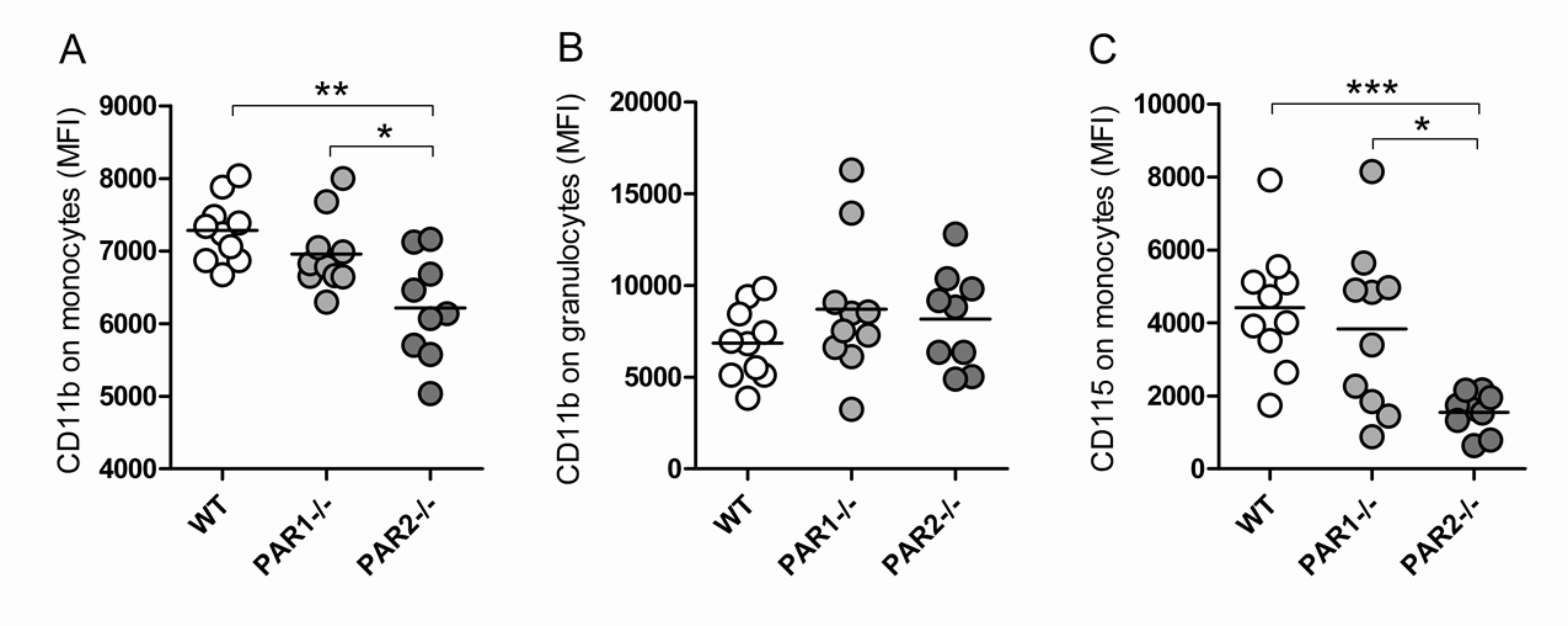

Supplement: Figure S4 — Expression of CD11b on monocytes and granulocytes and CD115 expression on monocytes in WT, PAR1-/- and PAR2-/- mice. FACS analysis was performed with peripheral blood from WT, PAR1-/- and PAR2-/- mice before ligation. The expression levels (MFI) are shown of (A) CD11b on monocytes (B) CD11b on granulocytes and (C) CD115 on monocytes. (TIF) [file pone.0061923.s004.tif]
